# Measuring Information-Transfer Delays

**DOI:** 10.1371/journal.pone.0055809

**Published:** 2013-02-28

**Authors:** Michael Wibral, Nicolae Pampu, Viola Priesemann, Felix Siebenhühner, Hannes Seiwert, Michael Lindner, Joseph T. Lizier, Raul Vicente

**Affiliations:** 1 MEG Unit, Brain Imaging Center, Goethe University, Frankfurt, Germany; 2 Center for Cognitive and Neural Studies (Coneural), Cluj-Napoca, Romania; 3 Frankfurt Institute for Advanced Studies (FIAS), Goethe University, Frankfurt, Germany; 4 Department of Physics, University of California Santa Barbara, Santa Barbara, California, United States of America; 5 Center for Economics and Neuroscience, Friedrich-Wilhelms University, Bonn, Germany; 6 Max Planck Institute for Mathematics in the Sciences, Leipzig, Germany; 7 CSIRO Information and Communication Technologies Centre, Marsfield, New South Wales, Australia; 8 Max-Planck Institute for Brain Research, Frankfurt, Germany; Wake Forest School of Medicine, United States of America

## Abstract

In complex networks such as gene networks, traffic systems or brain circuits it is important to understand how long it takes for the different parts of the network to effectively influence one another. In the brain, for example, axonal delays between brain areas can amount to several tens of milliseconds, adding an intrinsic component to any timing-based processing of information. Inferring neural interaction delays is thus needed to interpret the information transfer revealed by any analysis of directed interactions across brain structures. However, a robust estimation of interaction delays from neural activity faces several challenges if modeling assumptions on interaction mechanisms are wrong or cannot be made. Here, we propose a robust estimator for neuronal interaction delays rooted in an information-theoretic framework, which allows a model-free exploration of interactions. In particular, we extend transfer entropy to account for delayed source-target interactions, while crucially retaining the conditioning on the embedded target state at the immediately previous time step. We prove that this particular extension is indeed guaranteed to identify interaction delays between two coupled systems and is the only relevant option in keeping with Wiener’s principle of causality. We demonstrate the performance of our approach in detecting interaction delays on finite data by numerical simulations of stochastic and deterministic processes, as well as on local field potential recordings. We also show the ability of the extended transfer entropy to detect the presence of multiple delays, as well as feedback loops. While evaluated on neuroscience data, we expect the estimator to be useful in other fields dealing with network dynamics.

## Introduction

Many phenomena in the world around us, such as traffic systems, gene regulatory networks, neural circuits and the Internet can be best understood in terms of complex networks. Understanding such networks requires knowledge about the existence and direction of the interactions in the network. Often, the network function also depends on the interaction timing. For example, understanding of the railway system is incomplete if only the location of train tracks and the direction in which they are used is known. At least information on train travel times is necessary to catch a glimpse of how the network serves its purpose, and only a timetable enables one to use this network efficiently. As in this example, interaction delays may have a pivotal role in understanding the function of complex networks.

In neuroscience, interaction delays arise mainly due to propagation of action potentials (‘spikes’) along axonal processes and can amount to several tens of milliseconds. The presence of axonal delays is of particular importance for coordinated neural activity (e.g. synchronization, Hebbian learning) because they add an intrinsic component to the relative timing between spikes. For example, two neurons projecting to a downstream neuron will be observed to spike simultaneously by this downstream neuron only when their relative timing of spikes compensates the difference in their axonal delays and in the dendritic delays to the soma of the target neuron. Indeed, disruption of coordinated activity by the pathological modification of axonal delays is thought to account for some deficits in diseases such as multiple sclerosis [1], schizophrenia [2], and autism [3]. Thus, the estimation of both, interaction delays and interaction strengths from multichannel brain recordings are needed to better resolve the dynamic coordination between different areas. In this paper we propose an extension of an information-theoretic *functional*, transfer entropy, to determine both the information transfer and interaction delays between processes.

In the following, we review the key concepts of Wiener causality and transfer entropy, and describe the outline of the paper.

### Causality, Transfer Entropy, and the Estimation of Interaction Delays

Ideally, in explorative analyses both the presence of directed interactions between two physical systems, as well as their timing should be detected without any *a priori* knowledge of the coupled systems or their specific interaction mechanism, i.e. a model free analysis is required. To keep our analysis as model-free as possible, we assume that the coupled physical systems 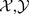 produce the observed time series 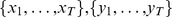 via measurements at discrete times 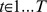. These time series are understood as realizations 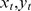 of stationary random processes 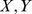 for mathematical treatment. The stationarity assumption for the random processes is convenient here as it allows to replace ensemble averages by time averages, but the proposed method will also work for ensemble averaging. In the remainder of the text, upper case letters 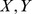 refer to these random processes, 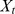,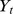 to the random variables the processes are composed of, while lower case letters with subscript indices 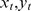 refer to scalar realizations of these random variables. Bold case letters 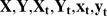 refer to the corresponding processes, random variables, and their realizations in a state space representation (see the methods section for the construction of these state spaces).

The structure of directed interactions can be analyzed by assigning a causal influence from a process 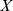 to another one 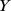, if knowledge about the past of realizations of 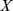 and 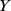 together allows one to predict the future of 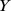 better than knowledge about the past of 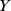 alone. This is known as Norbert Wiener’s principle of `causality’, and does not by itself entail a modeling approach. Although this principle paved the way for a formal analysis of directed interactions, we note that today for an analysis of truly causal interactions more stringent requirements have to be met [4], [5]. If only Wiener’s principle is met, we speak of *predictive information transfer* [6]. However, predictive information transfer may often be exactly the quantity of interest when analyzing directed interactions in networks, especially when these networks actively process information [6], [7].

Wiener’s principle can be directly translated into an information-theoretic framework by reformulating it as the question: “*What information does the past of 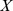 provide about the future of 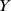, that the past of 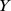 did not already provide*?”. Schreiber [8] formalized this question in terms of a conditional mutual information 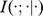 between the involved quantities:
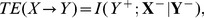
(1)where 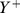 is a future random variable of the process 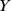, whereas 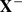 and 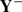 denote suitably reconstructed past *state variables* of the processes 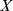 and 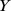, respectively. The corresponding quantity has been described several times in the literature (e.g. [8], [9]) and is most often refered to as *transfer entropy* [8].

The use of transfer entropy or related methods [10], [11] for model free analyses of directed interactions has seen a dramatic surge of interest recently, both in neuroscience [9], [12]–[31], physiology [32]–[34], as well as in the general theories of computation [7], [24], [35] and causality [5]. For specific application scenarios transfer entropy has proven to be clearly superior compared to alternative analyses of interactions [22].

Schreiber originally defined the transfer entropy functional for random processes 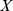 and 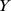, with discrete-valued time index 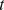, as [8]:
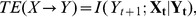
(2)


Yet, it was noted early on that in real world physical systems information from 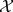 needs a finite time 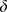 to arrive at 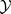. Because of this, past states variables 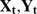 and future future random variables 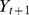, that replace the abstract quantities 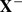,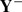 and 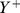 in the functional from equation 1 in the calculation of a specific estimator, such as in equation 2, have to be redefined appropriately to reflect this fact. Therefore, two suggestions were made to adapt transfer entropy:

- First, we and others suggested to use the following formula to take efficiently into account the possibility of a non-vanishing interaction delay [9], [12], [13], [29],
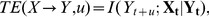
(3)where the parameter 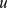 is the time which an influence needs to propagate from 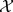 to 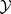 [9], [12], [13], [29]. A scanning approach for the parameter 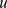 was suggested to recover the delay with the largest predictive information transfer and, thereby, recover the dominant interaction delay [12].
- Second, Pompe and Runge [36] suggested a similar scanning approach to adjust the TE to consider transfer from a previous source variable 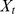 to a future target variable 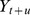, while conditioning on the source state variable 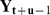, and additionally conditioning on the previous source state variable 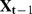:

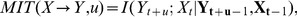
(4)This measure is known as the *momentary information transfer* (MIT) (see Methods section part on MIT for full details). While the idea to scan 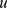 in order to maximize MIT is similar to the first approach, the conditioning on a past source state was thought to ensure that the specific delay was identified where the transferred information appeared in the source first.

While the first approach in equation 3 seems like a natural extension of transfer entropy, we will show in the next section that equation 3 violates Wiener’s principle of causality. This is because the above functional violates the requirement of an optimal self prediction from the past of 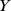 to the future of 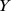 that is implicit in Wiener’s principle. In short, using a state 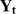 that is not obtained *immediately prior* to the future 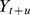 – the state one is trying to predict – ignores potentially relevant predictive information (see below for details). We will provide a simple example where the above functional (equation 3) from references [9], [12], [13], [29] does not recover the correct interaction delay.

We will also show by counter-example that the second suggestion, the MIT, is not able to reconstruct the correct interaction delay in a simple example in the presence of memory in the source.

Therefore, we present in this study an improved transfer entropy functional that honors the requirement of an optimal self prediction and that successfully recovers the correct interaction delay. We formulate a mathematical theorem that the improved transfer entropy functional is maximal when its delay parameter coincides with the underlying interaction delay and give the corresponding proof. To further validate our approach on finite data we run extensive simulations of stochastic and deterministic delay-coupled systems. Local field potentials are also used to test the recovery of interaction delays in electrophysiological recordings.

Furthermore, we will demonstrate below that our novel approach allows to test the presence of self-feedback activity in a single recorded signal. Finally, we discuss how information about interaction delays can be used to enhance the power of effective connectivity analyses.

## Results

### Comparison of Transfer Entropy Functionals in Relation to Wiener’s Principle

Wiener’s principle asks for information about the future of 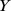 that the past of 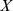 can provide *in addition* to the information already provided by the past of 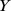. If, however, this latter information is underestimated, we may potentially obtain an erroneously high value of predictive information transfer from 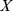 to 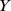. From this it follows that information provided by the past of 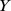 to the future of 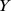 must be estimated optimally, a fact that we will refer to as the *self prediction optimality* (SPO) requirement from here on. Equivalently, from an information-theoretic computational perspective, we can view the ‘self prediction’ as an *information storage* by 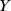 [37], and so underestimating the information storage in 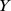
’s dynamics can lead us to overestimate the information transfer from 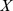 to 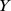 [7], [35].

For practical applications of Wiener’s principle this means that we have to guarantee an optimal self prediction at least within the limits of our respective prediction framework. For the most general case of TE where no a priori model-based knowledge can be used, this means we have to resort to model free prediction, as it is for example provided by the local predictor [38], [39].

We will first show that the transfer entropy functional given in equation 3 is not self prediction optimal and then provide a new transfer entropy functional that is self prediction optimal.

To show that the functional from equation 3 is indeed not self-prediction optimal, we rewrite this equation by substituting 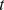 by 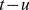:
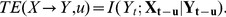
(5)


We see that the self prediction of 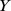, or equivalently the conditioning, is done based on the state 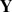 at time 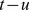. Let us define 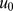 as the value of the parameter 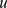 that optimizes self prediction of 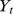 by 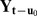. If we now scan 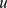 in search of the maximum predictive information transfer from 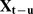 to 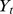, we will potentially condition our mutual information on states 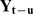 with 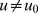, i.e. we condition on states that are not optimal for self prediction. This suboptimal conditioning may artificially inflate transfer entropy values in a 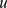-dependent manner. Thus, maximum transfer entropy values do not only depend on the true information flow from 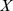 to 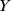 and its delay, but also on the quality of the self-prediction (conditioning). As a consequence, the maximal apparent information transfer estimated by equation 5 may be found at values of 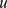 that do not represent the true delay 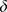, and the attempt to identify the true interaction delay by maximizing predictive information transfer with the functional in equation 5 may lead to erroneous results.

From the above it follows that conditioning on the past state 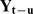 should always be done with respect to the optimal state 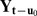. Hence, a modified functional reads:
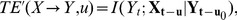
(6)where 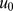 in principle would have to be identified by a model-free prediction scheme, such as the one proposed by Ragwitz and Kantz [39]. However, we can abbreviate this procedure and formally prove that 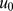 must be 1 sample if equation 6 is to represent a *causal relationship* (see next subsection). Furthermore, using 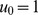 properly eliminates any *information storage* from the past of 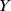 that could otherwise be mistaken as information transfer from 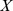. And finally, the use of 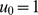 allows us to take a dynamical systems view of the *state transition* 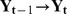, and consider the TE as measuring how much information 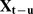 provides about this state transition.

#### Result

*The predictive information transfer from 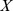 to 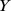 over a time delay 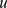 is properly captured (aligning with Wiener’s principle) by*:
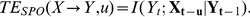
(7)


This functional fulfills the self prediction optimality requirement and we chose the subscript ‘SPO’ to reflect this. We note that in the case 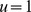 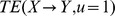 the old estimator (equation 3 ) is equal to 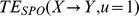 (and this equality also holds for the original formulation of the TE from equation 2 ); however, this does not hold for other 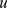 in general and as such using equation 6 with 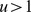 and 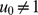 does not satisfy Wiener’s principle. To see this, we rewrite from equation 6 : 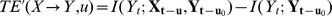, which allows us then to see explicitly that both, (1) the joint information term 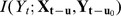 supplied by the source and the past of the target, and (2) the information storage term 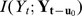, differ under the cases 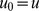 (old delayed TE estimator, equation 3 ) and 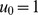 (new delay TE estimator, equation 7).

In the next section we provide a theorem that formally states that 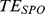 is maximal when the parameter 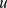 is equal to the true interaction delay 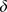, and give a proof. Thus, 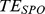 can be used to recover an interaction delay 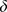 in coupled systems as:
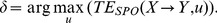
(8)


### A Theorem on the Identifiability of the True Delay 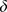 of an Interaction

Part of this study is a proof of the fact that the new proposed functional assumes its maximal value when the delay parameter 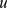 in 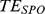 (equation 7) is equal to the true delay 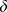. The main finding can be summarized in the following theorem:

#### Theorem 1

*For two discrete-time random processes 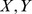 with a state space representation 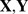, coupled from 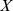 to 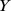 via a non-zero delay* 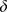, 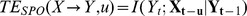 is maximal for 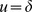. *This also holds in the presence of additional coupling from 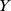 to* 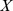.

**Figure 1:**
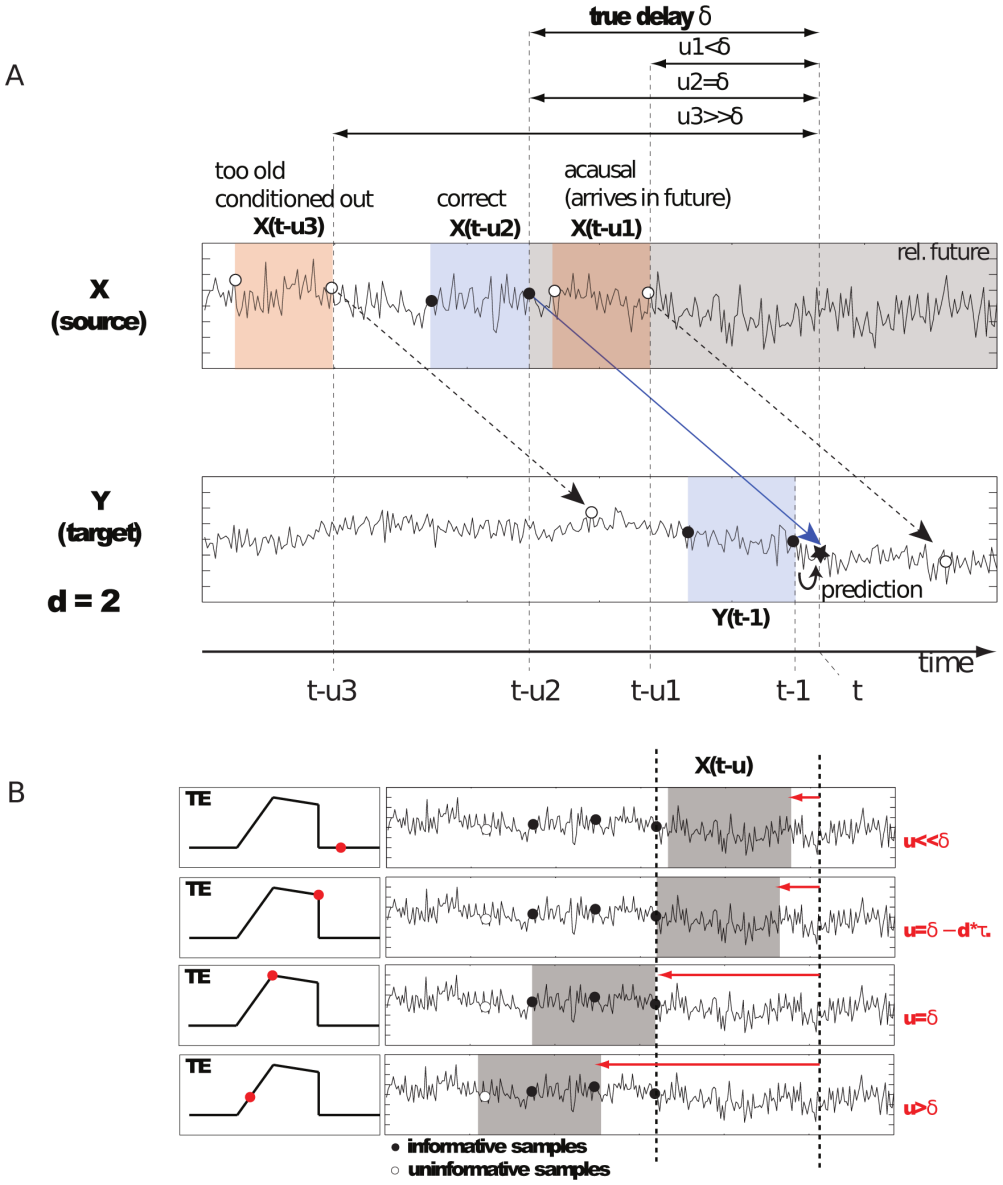
Illustration of the main ideas behind interaction delay reconstruction using the TE_SPO_ estimator. (A) Scalar time courses of processes 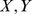 coupled 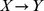 with delay 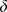, as indicated by the blue arrow. Colored boxes with circles indicate data belonging to a certain state of the respective process. The star on the 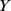 time series indicates the scalar observation 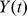 to be predicted in Wiener’s sense. Three settings for the delay parameter 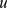 are depicted: (1) 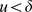 with – u is chosen such that influences of the state 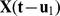 on 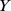 arrive in the future of the prediction point. Hence, the information in this state is useless and yields no transfer entropy. (2) 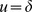 – u is chosen such that influences of the state 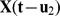 arrive exactly at the prediction point, and influence it. Information about this state is useful, and we obtain nonzero transfer entropy. (3) 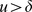 – u is chosen such that influences of the state 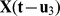 arrive in the far past of prediction point. This information is already available in the past of the states of 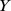 that we condition upon in 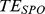 Information about this state is useless again, and we obtain zero transfer entropy. (B) Depiction of the same idea in a more detailed view, depicting states (gray boxes) of 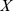 and the samples of the most informative state (black circles) and noninformative states (white circles). The the curve in the left column indicates the approximate dependency of 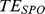 versus 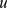. The red circles indicates the value obtained with the respectzive states on the right.

The main ideas behind delay reconstruction via maximizing 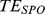 are illustrated in Figure 1. By scanning the delay parameter 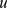 we shift the considered state 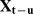 of the source process 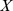 in time. If this state 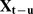 is in the relative future of the observation to be predicted for 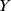, i.e. 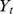, its influence has not arrived at 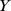 yet. As a consequence, the state is uninformative and we get low 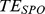. If the state has a time delay 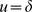, such that the influence arrives exactly at 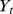, then 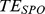 is maximal. If the state has too long a delay, then its influence has arrived before 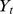 and is already taken into account via conditioning on the past state 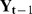; again we obtain low 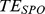. In the following we will present our proof. Since it is of a technical nature the reader may safely skip ahead if not interested in this material.

### Proof of Theorem 1

#### Outline

We start by showing that the three random variables 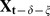, 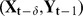, and 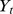 form a Markov chain for 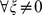. To this end we first demonstrate the *d-separation* of 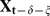 and 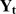 by 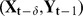 which is equivalent to conditional independence of 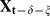 and 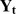, given 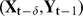 [4]. This, in turn, is equivalent to 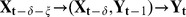 forming a Markov chain. Using the *decomposition property* of conditional independence (e.g. [4]), we see that this result also holds if we replace the state 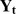 by the corresponding scalar observation 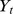. We then use this Markov chain for statements on the relation of mutual information terms built from its variables and rearrange terms to arrive at the statements on TE formulated in theorem 1.

#### d-separation

**Figure 2:**
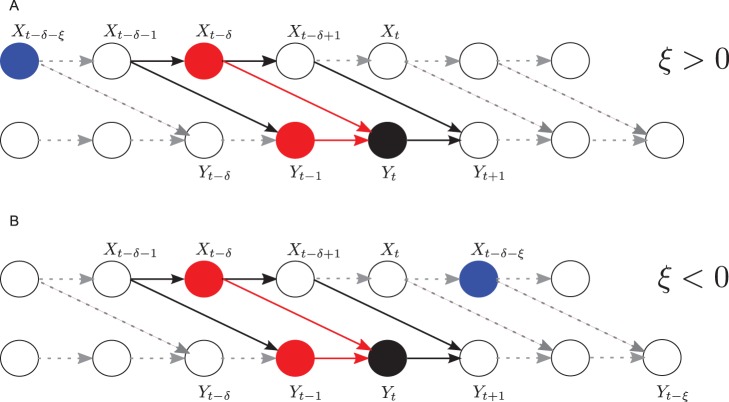
Causal graph for two coupled systems 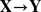. Illustration of d-separation of 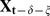 and 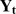 by 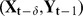. Arrows indicate a causal influence (directed interaction). Solid lines indicate a single time step, broken lines an arbitrary number of time steps. The black circle is the state to be predicted in Wiener’s sense, the red circles indicate the states that form its set of parents in the graphs. These states are also the ones conditioned upon in the novel estimator 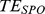. The blue circle indicates the state in the graph for which we want to determine that forms a Markov chain: 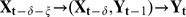. For 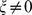 all sequential paths from 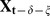 into 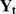 are blocked, as are the divergent paths between these nodes. All convergent paths (e.g. via 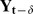 in (B)) are not blocked. This holds for 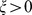 (A) and 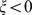 (B).

From figure 2, representing the causal graph of the two random processes 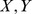, we see that:

- all *sequential* paths from 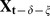 into 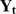 are blocked by the joint random variable 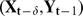 (sequential paths of this kind only exist for 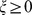, i.e. states of 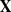 that are further back in time than the true interaction delay 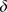.),
- whereas *none* of the *convergent* paths between 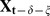 and 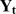 are blocked, because we do not condition on 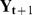 or any other future value of 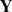,
- and all of the *divergent* paths between 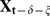 and 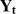 are blocked by conditioning on 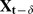.

Hence, 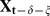 and 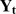 are indeed *d-separated* by 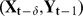.

#### Conditional probability distributions and Markov chain property

Given this *d-separation*, we can state for the corresponding conditional and joint probability distributions 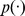 that:
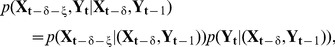
(9)which is equivalent to 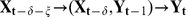 being a Markov chain. From this it follows via the *decomposition property* of conditional independence that the following is also a Markov chain: 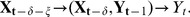
(10)


#### From the markov chain property to delay reconstruction

Building on equation 10 we see that the Markov property still holds if we form a new random variable from 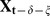 by considering it jointly with 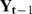, and we obtain:
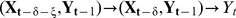
(11)


Using the data processing theorem, this leads to an inequality for mutual information terms between the variables as:
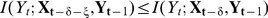
(12)


By subtracting the *active information storage* [37], 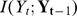, from both sides of this inequality we get:
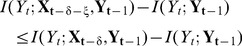
(13)and, by using the chain rule for conditional mutual information:


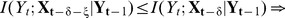
(14)


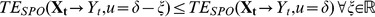
(15)

Hence, the value of 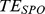 is indeed maximal when the parameter 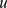 is equal to the true delay 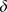 – q.e.d.

#### Remarks

Since the above derivation does not hold if we replace the conditioning set of variables 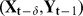 by 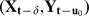 with 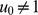 (because the relevant d-separation is not maintained), then we must set 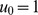 to obtain an estimator with a potential causal interpretation.

Similarly, we note that the use of 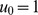 is necessary to eliminate information storage 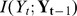 from the past of 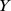 being attributed to the predictive information transfer 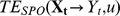.

Importantly, since this proof only relied on a proper conditioning of the probability distributions of the parents of 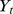 in the causal graph, it also holds for bidirectional coupling, as the parents of 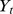 in the causal graph do not change by adding coupling from 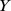 to 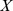.

We also note that some constraints apply: (a) If the two systems cannot be directly observed, but only noisy observations 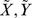 of the true systems dynamics are available, theorem 1 cannot be proven for these noisy observations 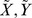, but may hold for many cases – see the examples given int the results section. We can also provide a proof (not given here) for autoregressive, linearly, unidirectionally coupled systems, where at most one of the two observations is noisy, i.e. we have 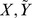. However, counter examples can be found for very pathological structures of the noise on the two systems, that are not expected in physical systems (multi modal combinations of 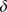-distributions). (b) Simulations show that for Gaussian noise of low amplitude, delays can still be identified correctly. (c) If the bidirectional coupling leads to full synchronization of the two systems, such that the probability distributions in equation 17 are delta distributions, transfer entropy is not defined. (d) There must be no other indirect sequential paths 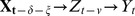 (for some 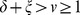) via some other variable 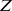 which are not blocked by 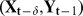 since this would violate the d-separation here. Extensions of the proof to this situation are conceivable, but are the topic of future studies.

### Inability of Momentary Information Transfer to Reconstruct Coupling Delay

In order to contrast 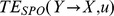 with the momentary information transfer (MIT) 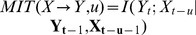 [36], we next examine two test cases. Case (Ia) contains noisy short-term source memory which leads to an erroneous delay estimation by MIT; in contrast, case (Ib) is an example that was reported to produce erroneous results in 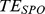 in [36], but we show here that this was due to the use of a symbolic preprocessing step in [36] and that 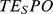 is perfectly capable to reconstruct the correct delay.

As explained in the methods section, in test case (Ia) 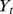 is a direct function of 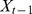, while 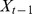 itself is a noisy mapping from 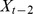 (with noise parameter 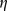, also see table 1 ). As such, 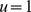 should be identified as the correct interaction delay here, although the source memory makes 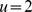 a potential candidate for an incorrect identification. The dynamics for test case (Ia) were run to provide 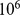 observations for estimating the required probability distribution functions. The estimation of the modified transfer entropy functional and the momentary information transfer was performed for this test case with the open-source “Java Information Dynamics Toolkit” [40] as detailed in the Methods section.

**Table 1:** Definition of stochastic self-mapping updates 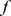 with memory and noise for variable 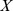 in test case (Ia).

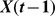	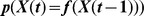			
	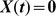	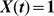	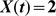	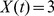
0	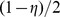	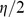	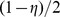	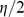
1	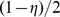	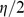	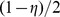	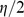
2	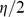	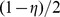	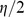	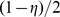
3	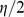	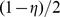	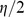	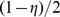

**Figure 3:**
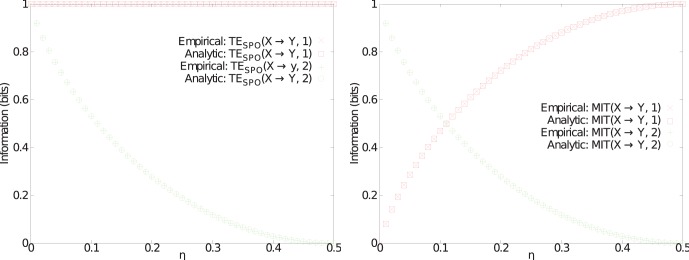
Test case (Ia), comparison of MIT and TE. Analytic and empirical measurements of (a) Transfer entropy 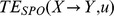 and (b) Momentary information transfer 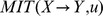 as a function of memory noise parameter 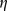 for the discrete-valued process with short-term source memory and a delay 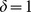. Each measure is plotted for delays 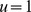 (red) and 2 (green). The correct causal interaction delay coorsponds 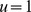 and therefore we expect an appropriate measure to always return a higher value with 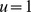 than with 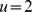, i.e the red curve should always be at higher values than the green curve. Nevertheless, there is potential for 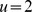 to be identified erroneously as the delay due to the presence of memory in the source 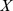, and MIT indeed finds this result for a range of the memory noise parameter 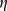 (below 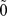.1).

Figure 3 shows the results of measuring 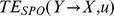 and 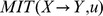, with delays 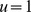 and 2, as a function of the source noise parameter 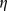. We see that, in line with our earlier proof regarding this situation of unidirectional coupling, 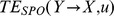 consistently identifies the correct delay 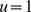, since 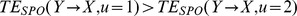 for all 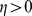. On the other hand, for a significant range of 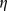, 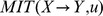 is deceived by the source memory into incorrectly identifying 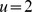 as the relevant delay.

Certainly 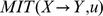 fulfills its design in identifying the lag to the time step of the source where the relevant information in the target first appeared. As we see in this example however, the existence of some information regarding the target variable in the source at a certain lag does not mean that this relevant information was transferred to the target at that particular lag. Here, the memory in the source makes the source strongly correlated to the target over lag 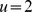. This drives both measures to high values for 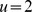 with low noise 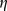, yet while 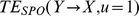 remains higher still, 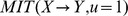 conditions out this correlated information and so falls below 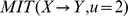. MIT returns the wrong result here because the conditioning on previous source state is not necessary and removes relevant information.

Finally, we examine test case (Ib), a bidirectionally coupled logistic map, for which Pompe and Runge [36] found that the 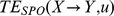 incorrectly identified the interaction lag as 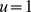 instead of 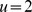, when this quantity was estimated using symbolic mapping (capturing ordinal relationships). In contrast to the findings in [36], our analysis described in the methods section using Kraskov-Grassberger-Stögbauer estimators [41] is able to accurately identify the correct lag 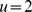 as having larger 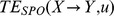 (2.123 bits for 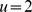 as compared to 0.826 bits for 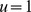). This result is in line with our proof that 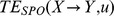 is maximized at the correct delay even in the case of bidirectional coupling. It is also in line with the more detailed empirical results we obtained for bidirectionally coupled processes as presented below. The opposite finding in [36] appears to simply be an artifact of their symbolic mapping approach: symbolic mapping may be a useful technique to handle small data sets, but it certainly removes parts of the information about the processes, and this information may well be relevant. Certainly, this is the case with coupled logistic maps, where examining ordinal relationships will miss many of the subtleties regarding how consecutive states are updated by the map.

### Estimating Interaction Delays from Simulated Data

Here, we test the capability of 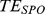 to detect the interaction delays from a series of simulated and experimental time series. The different cases cover stochastic, deterministic and real time series, representing different interaction configurations and delay ranges, and are described in detail in the methods section. The estimation of the modified transfer entropy functionals in these test cases (II-IX) was performed with the open-source MATLAB toolbox TRENTOOL [42] as detailed in the Methods section. State space reconstruction was performed using the Ragwitz criterion [39] in TRENTOOL, to obtain states that allow optimal self prediction, given the data. Throughout this section the *estimated* delays are indicated by 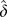, whereas the true, simulated delays are indicated by 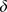.

#### Overview of simulated test cases II-IX

**Figure 4:**
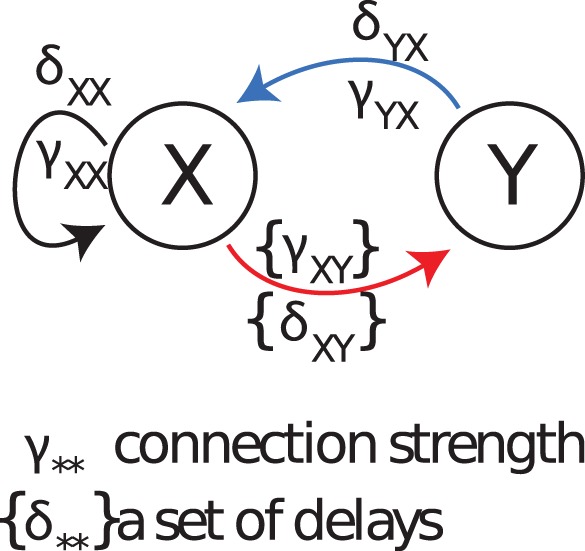
Overview over the structure of simulated test cases II-IX. Note that not all combination of links and parameters are always investigated. For details refer to table 2.

Figure 4 presents the general structure of test cases (II-VII,IX). All these cases comprise two systems labeled as 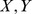, which are either both autoregressive order 10 processes (AR(10), equation 29), or both Lorenz systems (Lorenz, equation 32). For the Lorenz systems, the second coordinate (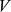 - see equation 32) was used as the observable producing the time series used for analysis. The systems may interact in the direction 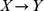, with either a single delay 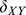, or a set of delays 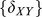, with coupling strengths 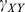 or 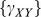, respectively. In the reverse direction 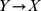 we only consider the case of single interactions with parameters 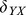, and 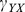. Additionally, in some of the cases delayed self feedback is present from process 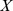 to process 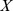, with delay 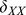, and strength 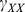. All simulated interactions, including self-feedback, were non-linear (quadratic) functions. One additional case (VIII) investigates delay reconstruction from a unidirectionally coupled ring structure of three Lorenz systems; the last case (IX) simulates the effects of observation noise on delay reconstruction. Details of the test cases are presented in table 2. For each test cases 50 data segments (trials) of 3000 sampling points each were simulated, resulting in a total of 150.000 data points. A full description of the generating equations for the system dynamics and the simulation details can be found in the subsection on the test cases in the methods section. In the following, we present results for these eight test cases (II-IX), with test case (V) serving as an example for the inability of the ‘old’ estimator [9], [12], [13], [29] to recover the correct interaction delays.

**Table 2:** Parameter settings used to create the simulated test cases II-IX.

Testcase	System	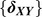	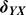	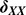	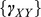	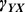	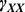
II	AR(10)	20	n.a.	n.a.	0.1	0	0
III	AR(10)	{15,20,25,30,35}	n.a.	n.a.	0.1	0	0
IV	AR(10)	{18,19,20,21,22}	n.a.	n.a.	0.1	0	0
V	Lorenz	45	75	n.a.	0.1	0.1	0
VI	Lorenz	75	n.a.	45	0.3	0	0.3
VII	Lorenz	45	n.a.	75	0.3	0	0.3
VIII	Lorenz (Ring)	20	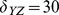	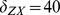	0.15	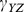 = 0.15	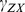 = 0.15
IX	Lorenz+Noise	45	75	n.a.	0.1	0.1	0

Lorenz = chaotic Lorenz system; AR = autoregressive processes. For the meaning of the coupling and delays constants 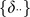 and 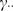 refer to figure 4.

#### Recovery of a single interaction delay

**Figure 5:**
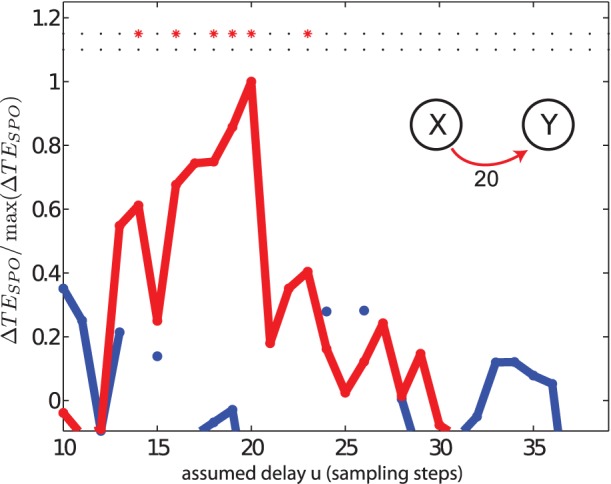
Test case (II). Transfer entropy (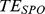) values and significance as a function of the assumed delay 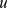 for two unidirectionally coupled autoregressive systems. For visualization purposes all values were normalized by the maximal value of the TE between the two systems, i.e. 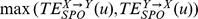. Red and blue color indicate normalized transfer entropy values and significances for interactions 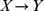 and 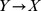, respectively. The nominal interaction delay 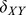 used for the generation of the data was 20 sampling units from the process 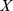 to 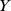. Asterisks indicate those values of 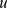 for which the p-value 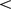 0.05 once corrected for multiple comparisons. Missing points for 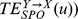 are because the analyses for these 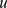
’s failed to pass the shift test (a conservative test in TRENTOOL to detect potential instantaneous cross-talk or shared noise between the two time series, see [42]).

In test case (II) we investigated two unidirectionally coupled autoregressive (AR) processes where a single interaction delay 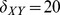 was present. We investigated 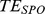 as a function of the assumed interaction delay 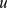. Figure 5 shows the results of computing 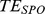 and its statistical significance (with a null hypothesis of no source-target coupling, see Methods) for the two possible directions of interaction, 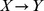 and 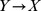. 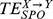 shows a maximal value for 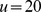 units, which matches the nominal value of 20 sampling steps. 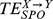 is statistically significant across a certain interval of delays around the maximum (14 to 23 sampling points) even when corrected for multiple comparisons. This blurring of the statistical significance of the predictive information transfer can be partly explained by *memory* in the source 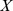 (via autoregressive terms) meaning the predictive value of the actual directly influential scalar observation 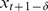 of the source is detectable in states 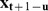 of 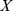 both *before and after* the actual delay 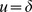 (compare the extension of sources states indicated by shaded boxes in figure 1). An additional factor here is that examination of the source states 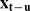 (instead of scalar observations 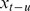) means that full information about the directly influential observation 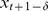 is contained in several source states *after* 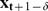. Crucially, the opposite direction (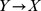) reveals a flat profile with no statistical significance, in correspondence with the absence of a directed interaction from process 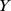 to 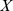.

#### Recovery of multiple interaction delays

**Figure 6:**
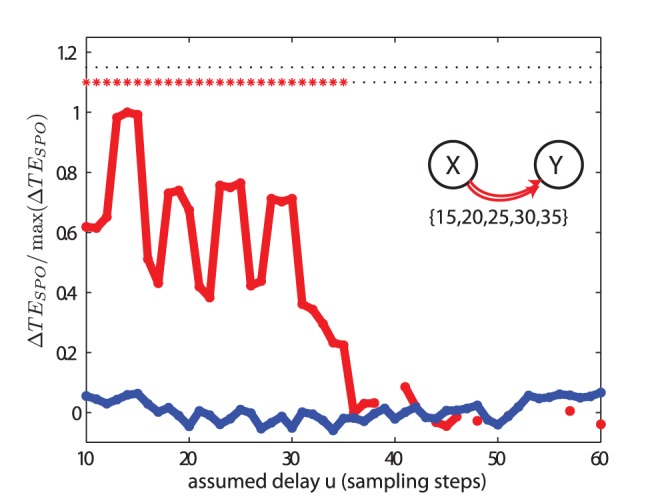
Test case (III). Transfer entropy (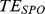) values and significance as a function of the assumed delay 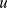 for two unidirectionally coupled autoregressive systems with multiple delays. The simulated delays 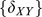 were 15, 20, 25, 30 and 35 sampling points. The rest of the parameters and criteria used are the same as those in Figure 5.

In the test case (III), we investigated two unidirectionally coupled AR processes where multiple interaction delays 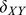 were present, 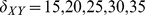. Figure 6 reveals that they can be readily detected by scanning 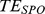. Well separated peaks indicate the presence of multiple delays around values of ∼ 14, 19, 25, and 30 sampling units for the direction of interaction 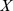 to 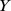. The curve displays an additional shoulder at 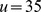. Nominal delays in the simulations were 15, 20, 25, 30 and 35, and thus all but the longest delay were correctly detected. The longest delay is most likely not detected because much information from the relevant source state 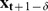 has already been communicated to the target over several shorter delays, due to the inherent memory of the AR(10) process, and there is no longer enough novel information provided by the source given the past state of the target to evoke a clear peak. However, the transfer entropy values 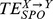 indeed were statistically significant up to an assumed delay of 35 units, in line with the maximal delay simulated.

**Figure 7:**
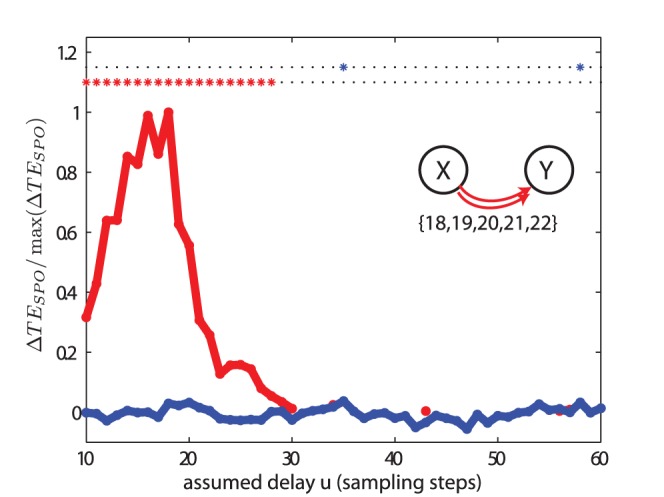
Test case (IV). Transfer entropy (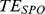) values and significance as a function of the assumed delay 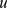 for two unidirectionally coupled autoregressive systems with multiple delays. The simulated delays 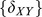 were 18, 19, 20, 21 and 22 sampling points. The rest of the parameters and criteria used are the same as those in Figure 5.

A more complex case (IV) is encountered when dealing with a smooth distribution of delays. Figure 7 demonstrates that in this case, a peak of 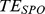 is attained near to the mean of the distribution of delays. The width of the peak is proportional to the width of the delay distribution. However, an exact estimation of the range of delays is difficult since single delays are also associated with broad peaks in the 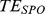 versus assumed delay curves (see figure 5, but note the different scale of the time axes). We note that the peak of 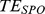 is skewed towards the shorter of the actual interaction delays, and this may be due to: (a) the longer delays providing less novel information from the source given that it is already contained in the target state from the shorter delays (as discussed in the above paragraph); and/or (b) the persistence of information of the current influential component 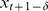 of the source state 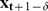 in several following source states (as discussed in the preceding subsection).

#### Recovery of delays of bidirectional interactions

**Figure 8:**
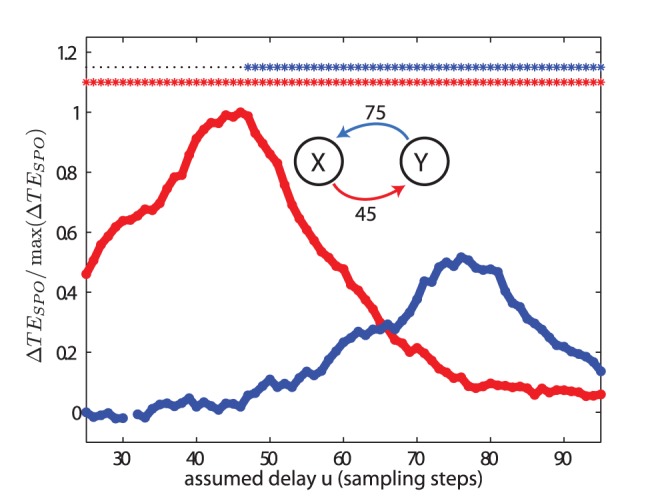
Test case (V). Transfer entropy (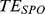) values and significance as a function of the assumed delay 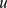 for two bidirectionally coupled, chaotic Lorenz systems. The simulated delays were 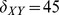 and 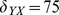, and the coupling constants were 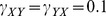. The delays were recovered as 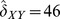 and 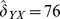. For more parameters see table 2.

**Figure 9:**
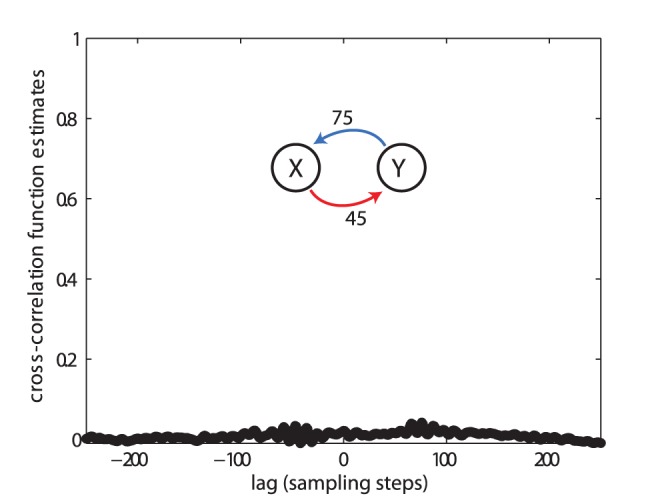
Test case (V). Crosscorrelation function for the two quadratically coupled chaotic Lorenz systems from figure 8.

For the analysis of two bidirectionally coupled Lorenz systems in test case (V), with 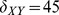 and 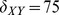, transfer entropy values peaked at 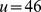 and 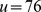 samples for the interaction from process 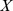 to 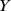, and 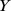 to 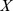, respectively (Figure 8). These values differed only by one sample from to the true interaction delays used for simulation. Moreover, the relation between the transfer entropy values for the two coupling directions reversed with increasing delay parameter 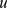: for delay values up to 65, transfer entropy values were larger for the direction from process 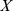 to 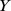, for values of 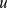 larger than 65 the opposite was the case. This is an important finding as the difference of the transfer entropies in both directions, also called the net transfer entropy, is often used as an indicator of the effective or dominating interaction structure. However, in our example, this net information transfer changed sign with changing delay parameter 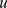. As an additional result, we show that the cross correlation function between the signals of the two systems was flat (Figure 9), as expected for a quadratic coupling.

#### Failure of the traditional estimator to recover the correct delays

**Figure 10:**
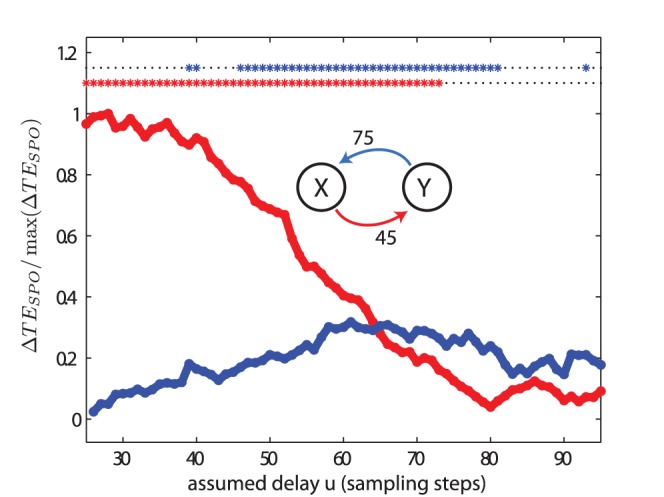
Test case (V) analyzed with the old estimator. Transfer entropy (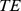) values and significance estimated by the old estimator from references [9], [12], [13], [29] as a function of the assumed delay 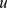 for two bidirectionally coupled, chaotic Lorenz systems. The simulated delays were 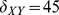 and 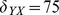. These delays were recovered erroneously as 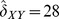 and 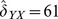. For more parameters see table 2.

We also analyzed the case (V) of bidirectionally coupled Lorenz systems with the ‘old’ estimator (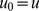) from references [9], [12], [13], [29] (figure 10). As expected on theoretical grounds, this type of estimator did not recover the simulated delays for the two coupling directions (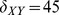 and 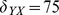), but instead delivered erroneous estimates (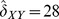 and 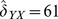).

#### Recovery of the delay of a feedback loop

We consider here the cases (VI) and (VII) where a feedback loop is affecting the dynamics of a node. In particular, we investigate first how the presence of feedback can be detected, and second how a feedback loop in a node affects estimation of transfer entropy to a different node.

We note that feedback loops do not pose a principal conceptual problem. Mathematically, a perfect state space reconstruction (see Methods for an explanation of state space reconstruction.) would subsume the feedback activity into the node’s dynamics. In practical terms, however, *long* self-feedback delays – in comparison to the intrinsic dynamics of the node – can not be covered practically in Taken’s classical state space reconstruction [43]. The reasons for this are twofold: (1) Self prediction performance may become unstable in high dimensional state spaces necessary to recover the delayed self-feedback. Algorithms determining optimal embedding parameters by optimal self prediction may get stuck in local minima this way. (2) Computational cost quickly diverges with a growing number of dimensions in state space. Hence, one may not even include the necessary number of dimensions and the necessary range of embedding delays in the parameter ranges that are searched to get the optimal embedding values. Given an imperfect state space reconstruction, even our new estimator is no longer self-prediction optimal, then. In the following we show that this estimator is nevertheless useful *to detect* self-feedback under conditions of long self-feedback delays.

**Figure 11:**
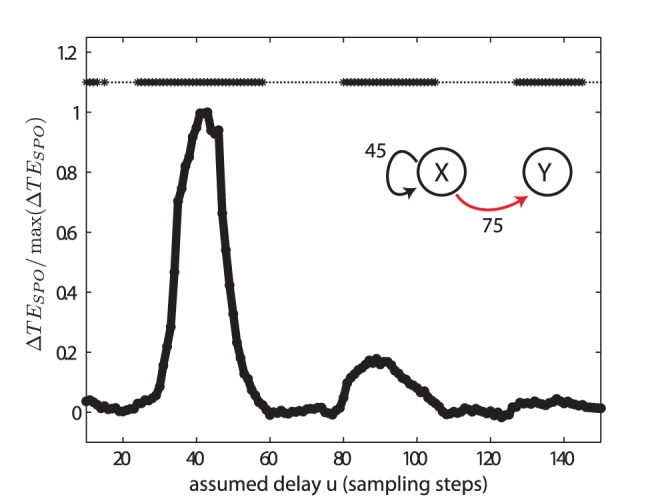
Test case (VI) - self-feedback analysis. Transfer entropy (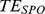) values between past and present of one of two Lorenz systems (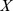) and significances as a function of the assumed delay 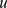. The analyzed chaotic Lorenz system was subject to a feedback loop with delay 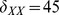, and an outgoing interaction 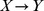 with delay 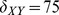, but no incoming interaction. The recovered delay for the self feedback was 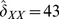, with a sidepeak at around two times this value. For the interaction analysis 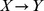 see figure 12. For more parameters see table 2.

Thus, we start by considering the dynamics of a single Lorenz system (process 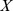 in test case (VI)) subject to a non-linear delayed feedback loop. This is, its own past output is fed back after it has undergone a nonlinear (quadratic) transformation (see the section ‘test cases’ in Methods). To detect the presence of feedback activity we evaluate 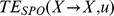 between the system’s original time series and its own past 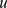 time steps back. To this end we choose embedding parameters such that the reconstructed states do not cover the feedback delay. The results are shown in Figure 11. Clear peaks are observed at 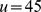, which corresponds to the simulated feedback delay 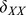, and its integer multiples. This corresponds to information being fed back via multiple rounds of the feedback loop.

The presence of feedback loops can challenge the reliable detection of information transfer between nodes. For example, if a node subject to feedback is unidirectionally coupled to another node, the direct computation of transfer entropy between the two nodes as a function of a delay parameter can lead to wrong inferences. To illustrate this point, we computed the self prediction optimized transfer entropy between the Lorenz system (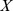) with a feedback loop and another Lorenz (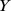) which receives its output.

**Figure 12:**
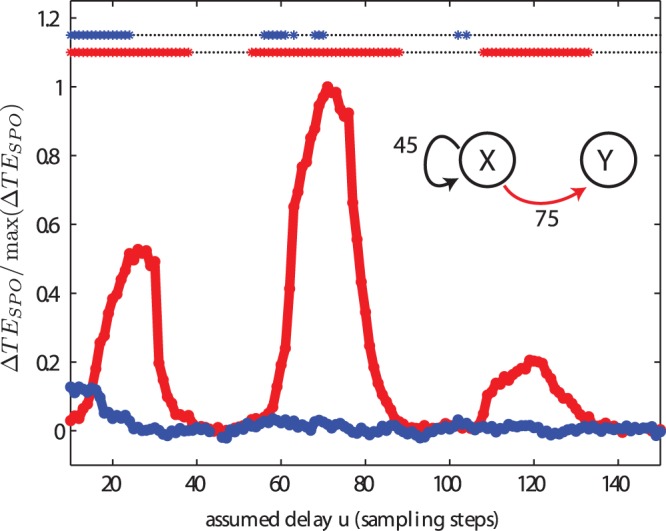
Test case (VI). Transfer entropy (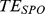) values and significance as a function of the assumed delay 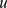 for a unidirectionally coupled chaotic Lorenz systems. The first Lorenz is subject to a feedback loop (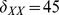) and unidirectionally couples to a second Lorenz with a interaction delay of 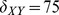 samples. Recovered delays were 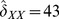 (see figure 11), and 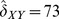. Sidepeaks were observed for 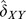 close to 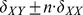. Spurious interactions were observed in the reversed direction at 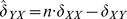, as it is expect for a system with self feedback [45]. Considering the positive test for self-feeback (figure 11) and the recovery of the self-feedback delay, the true system connectivity can be derived by combining the analysis of self-feedback and interaction delays.


First, we consider test case (VI) in which the feedback loop time (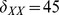) is shorter than the interaction delay between the systems (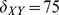). As shown in Figure 12, 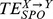 has the highest peak at 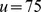, corresponding to the simulated interaction delay. However, it also shows clear peaks at 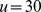 and 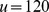, values which amount to the difference between the interaction and feedback delays, and their sum respectively. In general, peaks were observed at 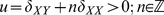, corresponding to different combinations of cycles around the feedback loop plus the interaction delay.

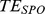
 from 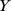 to 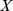, the direction in which no coupling was simulated, also exhibits several weak but significant peaks, i.e. we find false positive results in this case. Peaks were present at multiples of the delay feedback time minus the propagation time between the two Lorenz systems (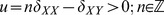). These peaks in 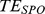 from 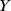 to 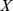 appear because the feedback loop in process 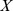 results in recurrent information in the dynamics of 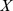 that can be predicted by the process 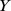, because thew process 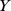 also receives a copy of them via the connection 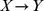. This information is useful to predict the state of 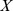 when the assumed delay 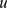 in 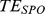 from 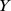 to 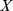 is at least as big as 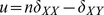, with 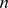 chosen such that 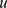 is positive. Notice that the size of the peaks decreases with larger 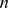.

**Figure 13:**
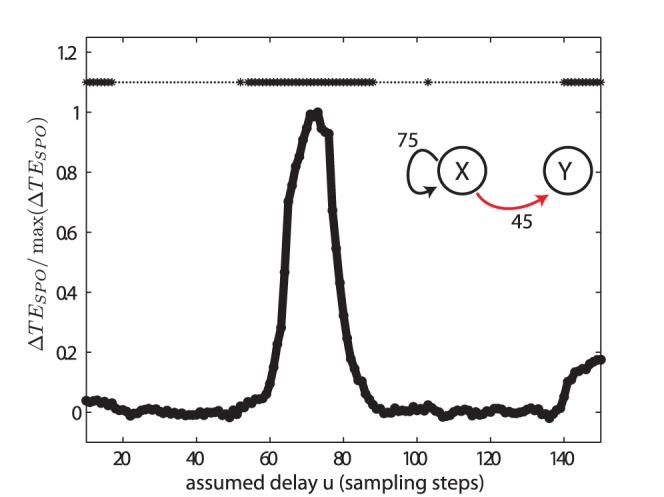
Test case (VII) - self-feedback analysis. Transfer entropy (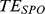) values between past and present of one of two Lorenz systems (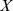) and their significances as a function of the assumed delay 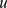 for a single chaotic Lorenz system subject to a feedback loop with delay 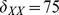, and an outgoing interaction 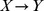 with delay 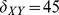. The recovered delay for the self feedback was 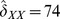, with a sidepeak at two times this value. For the interaction analysis 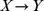 see figure 14. For more parameters see table 2.

**Figure 14:**
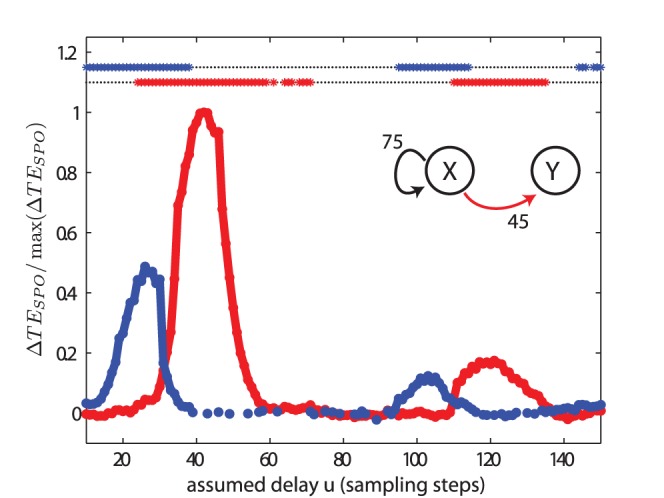
Test case (VII). Transfer entropy (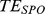) values and significance as a function of the assumed delay 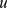 for a unidirectionally coupled chaotic Lorenz systems. The first Lorenz is subject to a feedback loop (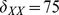) and unidirectionally couples to a second Lorenz with a interaction delay of 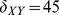 samples. Recovered delays were 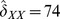 (see figure 13), and 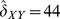. Sidepeaks were observed for 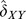 close to 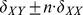. Spurious interactions were observed in the reversed direction at 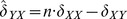, as it is expect for a system with self feedback [45]. Considering the positive test for self-feeback (figure 13) and the recovery of the self-feedback delay, the true system connectivity can be derived by combining the analysis of self-feedback and interaction delays.

Second, we considered test case (VII) in which the feedback delay 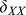 is longer than the interaction delay time 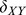 (see Figures 13 and 14). In this case structure similar to test case (VI) is observed for the location of the peaks of 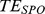. However, 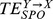 shows higher and more false positive peaks than in case (VI). This occurs since when 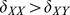, process 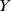 can predict the transitions that will occur in 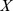 already after a single delay loop, because even for 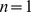 the condition 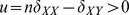 is fulfilled, – in contrast to a prediction of two delay loops ahead as in the previous case above. This situation is related to the so-called anticipative synchronization in which a slave system (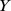) can anticipate the dynamics of the master system when this is subject to a long feedback loop [44], [45].

To be clear: these observations are not a theoretical problem with 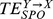 but are a practical issue in estimation (due to the impracticality of adequately forming Taken’s embedding of 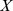 in the presence of the long delay loop, as described above).

#### Ring of Lorenz systems

**Figure 15:**
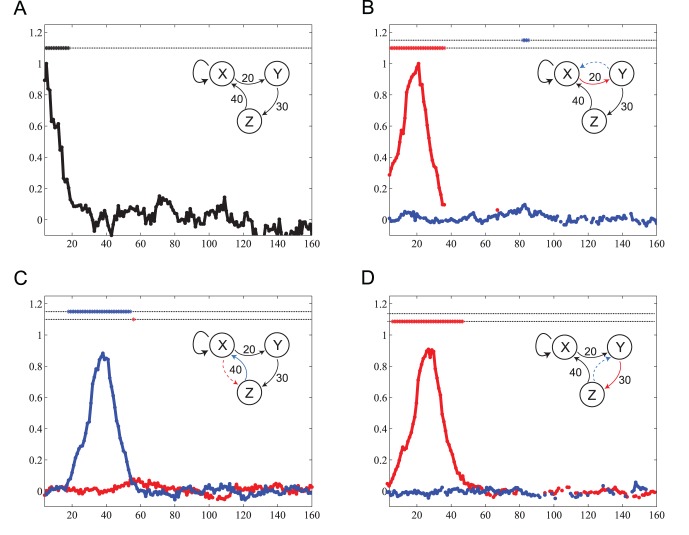
Test case VIII. Transfer entropy (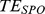) values and significance as a function of the assumed delay 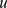 for three unidirectionally coupled chaotic Lorenz systems. The First Lorenz couples with the second Lorenz with an interaction delay of 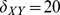 samples, the second Lorenz is unidirectionally coupled with the third Lorenz at a delay of 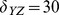 samples and the third Lorenz is unidirectionally coupled with the first Lorenz at an interaction delay of 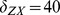 samples. The reconstruction of the simulated delays were: (A) self feedback, 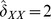, this value may be due to insufficient embedding, (B) 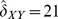, (C) 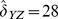, and (D)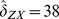.

In a network of three Lorenz systems coupled into a unidirectional ring, test case (VIII), our method identified the three simulated delays 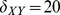, 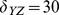, 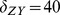 with reasonable precision as 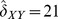, 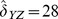, 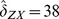 (figure 15). Analysis of self-feedback (as it is in principle present in a ring structure) for system 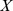 resulted in no significant peak at the expected sum of all three simulated delays (90), indicating that the information originally transfered from system 1 into the ring is *effectively* wiped out by the chaotic dynamics of the next nodes in the ring, a phenomenon well known in from coupled chaotic laser systems [46].

#### Effects of observation noise

**Figure 16:**
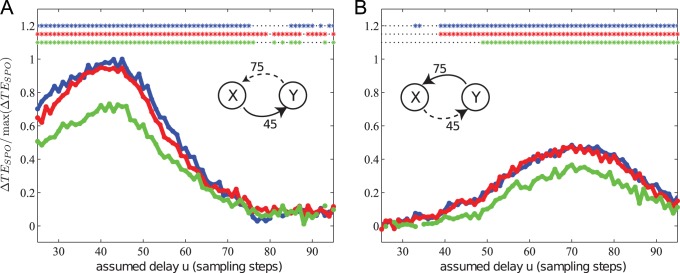
Test case IX. Transfer entropy (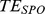) values and significance as a function of the assumed delay 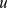 for two bidirectionally coupled, chaotic Lorenz systems. The simulated delays were 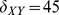 and 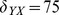. Observation noise with different amplitude was added to the simulated time series of the processes. The delays were recovered as (A) 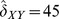 and 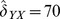 for 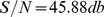 (blue), 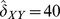 and (B) 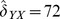 for 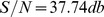 (red) and 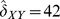 and 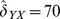 for 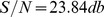 (green).

In test case (IX) we simulated two bidirectionally, quadratically coupled Lorenz systems with delays 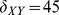, 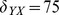, and added independent, Gaussian, white noise to the time series of the 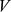-coordinate (see equations 32, 33 for details) before the reconstruction of delays. Observation noise did degrade the precision of delay reconstruction to a certain degree: with 1%, 2% and 9% of the total signal variance contributed by noise, the estimated delays were 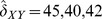, and 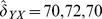 (figure 16). Note that noise amplitude and delay reconstruction error do not seem to be systematically related, suggesting that the effects of particular realizations of finite data cause the reconstruction errors.

### Local Field Potential Data

**Figure 17:**
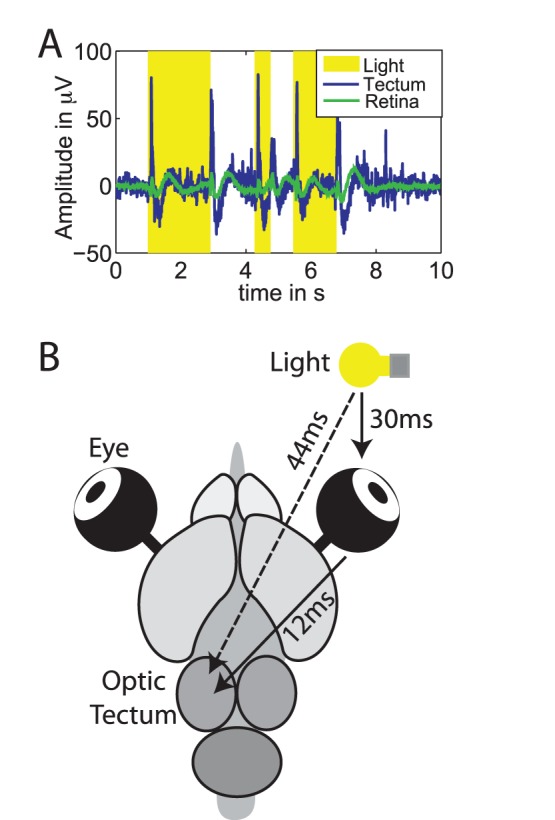
Interaction delay reconstruction in the turtle brain. (A) Electroretinogram (green), and LFP recordings (blue), light pulses are marked by yellow boxes. (B) Schematic depiction of stimulation and recording, including the investigated interactions and the identified delays.

To demonstrate that interaction delays can be reconstructed from biological time series with sufficient precision, we analyzed recordings of the electroretinogram (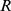) and local field potentials from the tectum (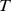) of the turtle brain (*Pseudemys scripta elegans*, figure 17). These data were recorded during stimulation (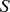) with light flashes at time points determined by a random process and with a duration drawn from a uniform random distribution; this ensured stationarity of the time series. In this experiment, direct physical interactions existed from the light source to the retina and from the retina to the tectum (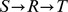). In addition, there was an indirect interaction from the light source to the tectum, mediated by the retina. This opens the possibility to check the precision of the delay reconstruction despite the fact the we have no precise knowledge of the true biological interaction delays. The evaluation of reconstruction precision is possible because the interaction delays reconstructed from 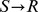 and from 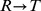 should sum up to the interaction delay reconstructed from 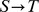 if reconstruction is precise.

We reconstructed the interaction delays the same way as in all previous test cases by scanning 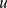. Indeed, the reconstructed delays were: 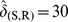
ms, 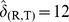
ms, 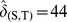 ms, meaning that the reconstructed delays between light source and retina and between retina and tectum added up to the reconstructed delay between light source and tectum with an error of 2 ms or 4.5%.

## Discussion

### Transfer Entropy Estimation without Violating Wiener’s Principle

We have laid out in the introduction why the earlier formulation of transfer entropy with an explicit time-delay as given in equation 3 and as used in [9], [12], [13], [29] is not a precise formulation of Wiener’s principle of causality in information-theoretic terms, as it violates the requirement of optimal self-prediction of the target time series. Accordingly, we were able to construct a test case were this functional gives a wrong estimate of the interaction delay in a system (figure 10), while the novel functional proposed here (equation 7), gives the correct result (figure 8). We therefore suggest to use this novel functional wherever interaction delays are expected. Accordingly, the new functional has been implemented in version 2.0 of our open source toolbox for transfer entropy estimation, TRENTOOL [42], and has been made the default option.

### Interaction Delay Reconstruction by Maximizing Predictive Information Transfer

In this study we demonstrated that it is possible to reconstruct the delays of interactions between two systems by finding the maximum of the predictive information transfer estimated by a novel transfer entropy functional, 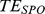, with a parametric dependence on the interaction delay. Our work complements earlier, anecdotal reports of delay-sensitivity of the predictive information transfer [12], [13], [42], by presenting a new formulation of the transfer entropy functional rigorously based on Wiener’s principle of causality and *backed by a formal proof* (see results section). Crucially, our experimental results identify the precise interaction delay for coupled systems with a single interaction delay, validating the formal proof which was constructed under these conditions. Furthermore though, we show that the reconstruction of interaction delays is possible for a large range of coupling types, multiple interaction delays, complex dynamics of the subsystems, for ring, and bidirectionally coupled systems. This last point is of great importance, as up to now, the analysis of bidirectionally coupled systems has often been discouraged, at least implicitly. Hesitation to analyze bidirectionally coupled systems is based on two observations - on the one hand, bidirectional coupling often leads to complete synchronization and in these cases an analysis of interactions is indeed not possible - on the other hand, it has been shown that the quantification of causal effect *size* is not always possible in these systems [47]. With our finding that in cases where there is no complete synchronization we can reconstruct at least the individual delays of bidirectional coupling (again backed by our formal proof), we hope to revive the analysis of such systems - that are abundant in nature and technology.

Formally identical functionals to 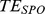 have been independently introduced several times in the literature, first by Nichols and colleagues [48], then by Overbey and Todd [49] – both in the field of structural integrity analysis of mechanical systems. In addition, Ito and colleagues used a formally identical functional to increase the detectability of interactions in spiking neural data [50]. In none of these studies the use of the functional for explicit delay reconstruction has been recognized and as a consequence no proofs for this property have been given. Ito and colleagues did indeed state that the delay parameter in their equation is there to account for finite delays, but they simply assumed maximality of the functional at the correct delay, without proof.

Note that the successful reconstruction of the true interaction delay between two coupled systems depends on reliable and precise enough estimates of the corresponding information-theoretic quantities (see methods section for the algorithms applied here). Obtaining these estimates may become a problem for small sample sizes. In this case, additional statistical testing against the null hypothesis of no coupling should be included when scanning delays and only maxima that show statistically significant coupling should be evaluated.

In addition, stationarity of the time series entering the analysis must be given, because transfer entropy between two random processes is typically defined via a time average that can only be used instead of the proper ensemble average for random processes if stationarity is given. If stationarity cannot be assumed *a priori* for the time series under investigation, appropriate testing should be performed (see [51], [52] and references therein). If at least cyclostationarity can be guaranteed, the proposed method could be used as functional within the ensemble averaging framework described in [53]. Reliably repeated non-stationarities may also be removed using the mathematical methods presented in [54]. In this case, however, additional testing for remaining non-stationarities is recommended. Note that for the local field potential data analyzed here, approximate stationarity was guaranteed by stimulation at multiple randomly chosen time points within each epoch of the experiment time series (i.e. “trial”), with stimulation durations also drawn from a random distribution.

### Comparison to the Momentary Information Transfer

As detailed in the methods section, *momentary information transfer* (MIT) [36] is an alternate approach to reconstructing the interaction delay, again using a scanning approach to maximize MIT as a function of delay 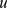. Both 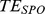 and MIT condition on the immediately previous target state (which is correct in comparison to the old TE estimator), and given that 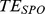 uses an embedded source state 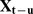, both measures include *synergistic* information that is jointly contributed by 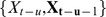 (as opposed to TE computed using only a single source *observation*). Yet a stark difference is the extra conditioning by MIT on 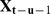, which removes *redundant* information that was already contained in the source. This prevents any of this information from being attributed to transfer at the lag 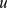.

A major conceptual difference between the Pompe and Runge study and ours is that no formal proof of the maximality of their functional MIT at the correct interaction delay is given, and as we argue below cannot be given.

Indeed, we provide a counter-example here – in test case (Ia) – where the momentary information transfer is not necessarily maximized at the correct interaction delay, in direct contrast to our proof of this property for 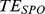. As such, MIT is not always reliably inferring the correct interaction delay.

The intention of the MIT in removing the self-redundant information in the source was to find the delay at which the relevant information about the target’s state update first appeared in the source. However, the availability of such information in the source at a specific time point does not mean that it is being transferred at that instant, and the presence of *memory* in the source inevitably leads the MIT to underestimate the influence of the source at the actual interaction delay (because of the removal of *redundant*, though potentially transferred, information). We demonstrated for these reasons that the MIT failed to identify the correct interaction delay in test case (Ia) on a simple, unidirectional coupling, single-interaction delay example with source memory, whereas our 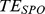 estimator functioned correctly.

As argued in [55], when investigating effective connectivity, the removal of redundant information by prematurely conditioning on other potential sources reduces our ability to infer connections. Conditioning on the source at another delay(s) should only be performed once one has already established the primary (or additional) coupling delay (see further discussion below and in [34], [55]).

Furthermore, the actual MIT analysis in [36] was carried out on a symbolic mapping of the time series (to vectors capturing the ordinal relationships between the variables). This approach, while potentially useful for short time-series realizations in magnifying certain relationships in the data, removes information on absolute values of the variables involved. While this loss of information may be harmless in climatology – the field of research MIT was aimed at –, it might be crucial to retain this information in other fields, such as neuroscience. Indeed, for the example in their study ([36], Section V.A) where the 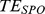 was not able to infer the correct interaction delay but 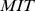 was, we have shown in test case (Ib) that this result is an artifact of the symbolic mapping in [36] rather than the measures themselves. Specifically, when we estimated in the continuous domain (retaining much information that the symbolic mapping removed), 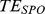 correctly identifies the interaction lag in this coupled logistic map process.

### Interaction Delay Fingerprints in Systems with Self-feedback

The phenomenon of self feedback is present in many complex networks. This self feedback can arise genuinely in the nodes of the network, e.g. by mechanisms such as autapses in neural networks [56], or because the systems receives (transformed) self-feedback via an unobserved part of the network. The analysis of interaction delays via 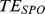 may offer valuable hints with respect to the presence of absence of relevant self-feedback.

Here we showed that 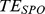 can detect the presence of feedback loops when applied to the time series of a single system and its own past – even if the system is chaotic and the feedback loops entail nonlinear transformations of the systems output. The information on the delay of a feedback loop can then be used to disentangle the potentially complex delay fingerprints, consisting of multiple peaks, that arise if such a system with self-feedback is coupled to other systems.

In principle, the multiple spurious peaks in the direction 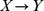 in our example, and all of the peaks in the direction 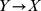 should vanish, given a state space reconstruction with states long enough to cover the delay feedback time. However, very long reconstruction lengths might lead to instable estimates due to the ‘curse of dimensionality’ [57]. In such a case, where the practically feasible reconstruction lengths for states are too short to cover the full dynamics of a system, the identification of feedback loops by 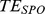 helps to better interpret the estimated pairwise information transfers between the nodes of a network. Time points around the self-feedback delay could in addition be included into the embedding states of a *non-uniform*, data-efficient embedding scheme [34], which would assist in removal of information storage and more accurate assessment of the transfer entropy as per the principles outlined in subsection on Wiener’s principle above.


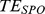
 differs from a simple application of the lagged auto-mutual information functional (AMIF) in that active information storage 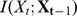 [37] contained in the most recent reconstructed state 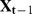 is removed. This will accentuate the presence of peaks in the delay spectrum compared to AMIF.

### Information Transfer Delays from Noisy Time Series

The proof for the identifiability of the true delay in the information transfer between two time series holds strictly only for the case of zero *observation* noise. Indeed the fundamental differences between time series obtained from Markov systems and those obtained from hidden Markov systems (i.e. from noisy observations) make it difficult to extend our proof without specifying the noise explicitly in each case. Nevertheless, our simulations of typical noise influences – such as independent, Gaussian, white noise – show that our approach works well in practice. While noise does indeed degrade precision, the random relation between observation noise amplitude and delay reconstruction error suggest that these errors are due to the combined effects of finite data and noise, and can be alleviated by increasing sample size.

### Relation to Linear Granger Causality and Corresponding Time Delay Reconstruction Procedures

Recently, Barnett and colleagues [19] demonstrated that transfer entropy and linear Granger causality are equivalent for the case of data with a Gaussian distribution. This result greatly simplifies the computation of interactions for data of this class. Neural data, however, do most likely not have a Gaussian distribution. This can for example be seen when comparing brain electrical source signals from physical inverse methods with the time course of corresponding ICA components [58]. Given that ICA components are as non-Gaussian as possible by definition, and given the fact that ICA components and brain source signals extracted by inverse methods closely match, we can interpret this as evidence for a non-Gaussian nature of brain signals. For these signals, TE may have an advantage for the analysis of directed interactions. On the other hand, the methodology presented here should be transferable to the domain of linear Granger causality in Gaussian data by virtue of the proof by Barrett and colleagues. Hence, the approach presented here may be seen as an alternative to earlier attempts to infer timing delays via linear Granger causality by inspecting time-dependent model-coefficients and using large model orders of 200 and more. For Gaussian data the scanning approach presented here would be equivalent to setting the first 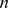 model coefficients to zero without having to estimate them, and scanning 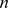, estimating the 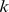 next model coefficients, where 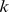 is the embedding dimension, resulting from optimization via the Ragwitz criterion [39].

### Relation of Delay Reconstruction and Multivariate TE Analyses

In systems composed of more than two interacting subsystems a pairwise, bivariate transfer entropy analysis as given by equation 7 may lead to wrong inferences with respect to the presence of an interaction between two subsystems. This happens when either a third subsystem drives the two subsystems under investigation with differential delays (‘common drive’), or when the two subsystems under investigation are connected indirectly via a third system acting as a relay (‘cascade effect’). The *potential presence* of interaction configurations of this kind can be detected by looking at timing relations across the graph of bivariate interactions. Here, this was demonstrated by our reconstruction of delays in LFP data in the turtle, where the interaction delay between light source and tectum was equal to the sum of delays on a route connecting the same end points (light source to tectum, via the retina). While both a common drive (light source 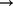 retina, light source 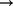 tectum) or cascade scenario (light source 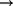 retina 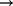 tectum) could explain the manifestations of these delays, what is important here is that this result is consistent with the known biological indirect interaction via the retina. If the concern is just to avoid false positive detection of interactions, a simple delay analysis approach may be a data efficient alternative to fully multivariate treatments of TE [7], [34], [35], [55]. That said, such multivariate treatments could yield further insights, e.g. if the *conditional* TE [7], [35] from the retina to the tectum conditioned on the light source (with appropriate delays incorporated) were statistically non-zero, then this would eliminate the possibility that the inferred retina 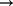 tectum relationship was a result of common drive by the light source. Combining delay analysis with multivariate treatments is feasible but more complicated (delays must be determined in an appropriate order, in the same way that the self-conditioning delay 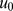 was determined here before the source delay 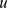 was explored - see some relevant discussion in [34], [55]); and will be the subject of a future publication.

### Delay Estimation Versus Significance Testing

We would like to stress here that inference on the presence of information transfer is a task separate from reconstructing the delay of the information transfer. For the former task we employed nonparametric permutation testing of 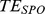 against surrogate data obtained from exchanging data epochs in the source. For the latter we searched the peak of 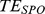. Our simulation results demonstrate that the existence of information transfer can typically be assessed over a wide range of assumed delays. On the one hand, this fact underlines the robustness of the 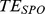-functional against misspecification of the delay parameter. On the other hand it is a warning not to conclude from the presence of significant information transfer at a certain delay parameter that the true delay is close to the assumed delay, if no scanning of the delay parameter was performed.

### A Practical Note on the Combination of Delay Reconstruction and Shift Testing

In practical applications, linear mixing or instantaneous crosstalk between signals occurs and may bias interaction measures based on Wiener’s principle [12], [59]. To detect such crosstalk, we have proposed a so-called shift-test [12], [13], [42]. This test determines if shifting the source time series into the past by the time that represents the assumed delay, 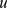, increases TE. This way two predictive information transfer terms get compared: on the one hand the predictive information transfer from the past of the source to the target, on the other hand the instantaneous information transfer from source to target. This procedure works very well as demonstrated in [42], but gives rise to minor a technical problem for real world data, because in these data instantaneous cross-talk is never truly zero. For assumed delays 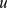 that are much larger than the true delay, the information transfer will be arbitrarily small, due to the combined effects of conditioning on the past of the target and the finite memory of the source. In contrast, cross-talk will always be non-zero. Hence, in situations with some finite cross-talk, there will a largest delay parameter 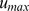, beyond which the presence of crosstalk will always be reported by the shift test. This, however, can be easily seen by comparing TE values at large 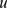 to those at smaller 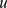. In contrast to a mere nuisance effect, relevant cross-talk should outweigh 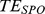 even at the optimal delay.

### Conclusion

We present a novel transfer entropy functional, which is a rigorous formulation of Wiener’s principle of causality in information-theoretic terms, respecting the condition of optimal self-prediction of the target time series from its own past. This functional has an explicit parametric representation of interaction delays between interacting systems. Scanning this parameter in search of the maximal predictive information transfer allows one to reconstruct interaction delays from a wide variety of systems.

## Methods

### Ethics Statement

Local field potential data were taken from experiments published elsewhere [42]. These animal experiments were approved by the German local authorities (Regierungspraesidium, Hessen, Darmstadt).

### Practical Transfer Entropy Estimation

In this section we outline the particular estimator of the 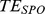 functional as provided in our toolbox TRENTOOL [42], and used in all analysis in this study. This realization relies on three steps: (1) state space reconstruction from scalar time series, (2) reformulation of the conditional mutual information in terms of four Shannon entropies, and (3) subsequent entropy estimation by a modified Kraskov-Stoegbauer-Grassberger estimator [41], [42].

As causality and interactions are defined as properties of systems, not scalar time series, we first have to reconstruct the corresponding state space of the interacting systems from the scalar time series. For this purpose we used Takens delay embedding [43] and optimized embedding parameters (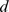 and 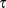, see below) according to Ragwitz’ criterion [39] for the target signal of each interaction pair. The use of Ragwitz’ criterion yields delay embedding states that provide optimal self prediction for a large class of systems, either deterministic or stochastic in nature. Delay embedding states of the systems under investigation can be written as delay vectors of the form:
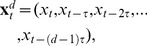
(16)where 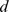 and 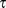 denote the embedding dimension and Taken’s embedding delay, respectively.

Using the states obtained by delay embedding we can rewrite transfer entropy as:
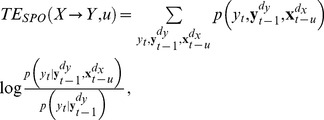
(17)or, using a representation in the form of four Shannon (differential) entropies, as:



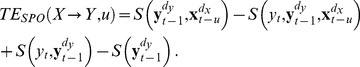
(18)Thus, 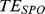 estimation amounts to computing a combination of different joint and marginal differential entropies. Shannon differential entropies can be estimated by nearest-neighbor techniques that exploit the statistics of distances between neighboring data points in a given embedding space in a data efficient way. This efficiency is necessary to estimate entropies in high-dimensional spaces from limited real data [60], [61]. Nearest-neighbor estimators are as local as possible given the available data. The assumption behind nearest-neighbor estimators is only a certain smoothness of the underlying probability distribution. Nearest-neighbor estimators can therefore be considered as non-parametric techniques, as desired for a model-free approach to transfer entropy estimation. Unfortunately, it is problematic to estimate TE by simply applying a nearest-neighbor estimator (e.g. Kozachenko-Leonenko estimator) separately to each of the terms appearing in equation 5. The reason is that the dimensionality of the spaces involved in equation 18 can differ largely across terms. Thus, fixing a given number of neighbors for the search will set very different spatial scales (range of distances) for each term. Since the error bias of each term is dependent on these scales, the errors would not cancel each other but accumulate. We therefore used the Kraskov-Grassberger-Stögbauer estimator which handles this problem by only fixing the number of neighbors 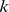 in the highest dimensional space and by projecting the resulting distances to the lower dimensional spaces as the range to look for neighbors there [41]. After adapting this technique to the TE formula [53], the estimator we use can be written as
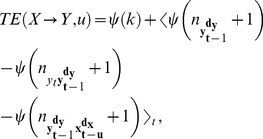
(19)where 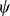 denotes the digamma function, while the angle brackets (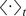) indicate an averaging over different time points. The distances to the 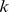-th nearest neighbor in the highest dimensional space (spanned by 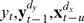) define the radius of the spheres for the counting of the number of points 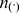 in these spheres around each state vector in all the marginal spaces 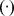 involved.

### Non-parametric Statistical Testing Against Surrogate Data

Even using Kraskov’s kernel estimation techniques as described above does not guarantee zero bias of the resulting estimator. Thus, the obtained TE values have to be compared against suitable surrogate data using non-parametric statistical testing to infer the presence or absence of directed interactions [12]. In short, the surrogate data must be produced under a null hypothesis of no source-target coupling, while retaining as many other statistical properties as possible (in particular the state transition probabilities 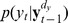). To this end we simulated and recorded data in an epoch based way and constructed surrogate data by shifting the time series of one of the two signals of each pair by one epoch, trying to preserve as many data features as possible (see detailed descriptions of the statistical routines in [42]). TE values were quantified as excess TE values with respect to surrogate data:
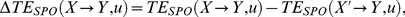
(20)where 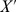 denotes the surrogate data. With respect to these surrogate data we also obtained significance values using permutation testing against suitable surrogate data as detailed in [42], to minimize the potential effects of bias introduced by noise and small sample size. Note that we assess statistical significance and quantify the excess transfer entropy 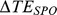 in both possible directions of interactions (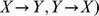, although interactions in some cases were unidirectional. By testing both directions nevertheless, we can also characterize the behavior of the proposed estimator with respect to false positive detection of interactions.

### Momentary Information Transfer

Pompe and Runge [36] recently proposed to reconstruct interaction delays using an information-theoretic functional, called *momentary information transfer (MIT)*. In their functional the interaction delay between two systems is also introduced in the form of a parameter of a conditional mutual information term. As for 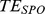 this parameter is scanned in order to maximize the value of their functional, 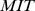. In contrast to our approach, conditioning of the mutual information in the method of Pompe and Runge is done with respect to the joint history of the *two* variables in question:
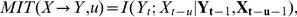
(21)


That is, while it retains the conditioning on the immediately previous state of the target 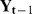 that we use in 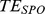, it *additionally* conditions on the state of the source 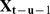 previous to the scalar source observation under consideration. The essence of Pompe and Runge’s argument is that their conditioning on 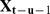 seeks to find the delay over which the transferred information is first available in the source, though we note that, as explored in earlier sections, the availability of such information in the source does not equate to it being transferred at that point. We note, that for our study, MIT was measured using discrete probability distribution functions, not the symbolic mapping (capturing ordinal relationships) that it is often associated with in the case of continuous data.

### Test Cases

We used simulated data and electrophysiological recordings to test the ability of the described methodology to detect interaction delays. The generation and characteristics of the studied time series are described below; note that we chose a bracket (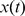) instead of a subscript notation (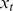) for time dependencies for this section to avoid cluttered subscripts. All analyses were performed in TRENTOOL (version 2.0.3, [42]) unless otherwise noted. The Ragwitz criterion [39] was used to determine the embedding dimension 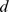 and lag 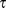. We used a significance level of 0.05, and corrected for multiple comparisons via false discovery rate (FDR [62]), to assess significance of the coupling. To identify interaction delays, we scanned the source delay parameter 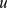 from 10 up to 150 time steps in steps of 1 sample.

#### Discrete-value process with short-term source memory

Test case (Ia) is formed from coupled discrete-valued processes 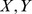, where 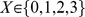 and 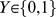, which were generated according to the equations:
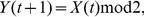
(22)

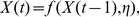
(23)where 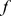 is a noisy self-mapping of 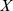 to its next value defined in Table 1 with noise parameter 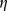. Note that 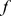 incorporates some randomness and some stochastic short-term memory in the next state. The update function 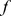 can be explained very simply if we consider 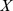 as a joint variable of upper and lower bits 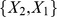, and understand that 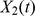 is randomly determined at each time step, while 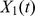 is a copy of 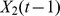 with probability 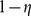, otherwise it is the inverse of 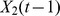. Using this interpretation, we have 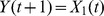.

Here, the true causal delay 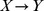 is 1 time step, though the source memory in 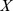 means that 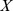 and 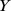 will be strongly correlated over a 2 time step delay also. In this case, we only examined these two candidate delays. Clearly, the Ragwitz criterion is satisfied here with embedding dimension 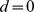 (there is no auto-correlation between values of 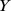).

As this system is discrete valued, MIT was measured using discrete probability distribution functions here, not the symbolic mapping (capturing ordinal relationships) that it is often associated with for continuous data.

#### Bidirectionally coupled logistic map

Test case (Ib) is taken from Section V.A of [36]. It is formed from the bidirectionally coupled logistic map processes 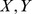, where we have 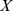 and 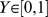, which were generated according to the equations:
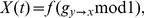
(24)

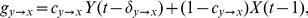
(25)

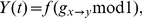
(26)

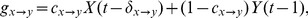
(27)

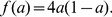
(28)


The process is run using the same parameters as in [36]: 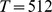 samples, 1000 repeated trials (results averaged over trials), 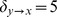, 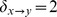, 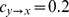 and 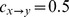. We run the system from random initial states and discard 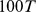 samples before collecting 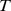 observations for our measures, to ensure the removal of transient effects. The embedding dimension 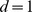 and lag 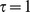 are selected to match those used in [36].

We examine 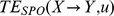 for delays 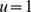 and 2; clearly the correct delay should be measured as 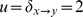. All calculations for test cases (Ia) and (Ib) were made using the open-source “Java Information Dynamics Toolkit” [40]; the results here can be reproduced using the demos/octave/DetectingInteractionLags demo of this toolkit. TE was measured using a Kraskov-Grassberger-Stögbauer estimator here, to contrast the results with those obtained for TE from symbolic mapping (capturing ordinal relationships) in [36] (which incorrectly inferred 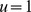 as the interaction delay).

#### Autoregressive (AR) processes

Coupled autoregressive processes 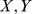 were generated according to the equations:
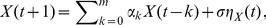
(29)

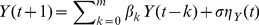
(30)

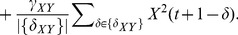
(31)where m = 10 is the order of the autoregressive processes, 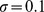 is the dynamic noise amplitude of uncorrelated, unit-variance, zero-mean Gaussian noise terms 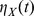 and 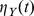, 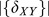 denotes the number of elements in the set of delays 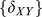, and specific values for the delays (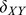) and coupling strengths (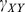) are listed in table 2 for test cases II-IV; the values for 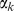 and 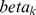 where constructed from roots of the characteristic polynomial of the the AR process, that were chosen at random on the unit circle to guarantee a stationary AR process.

#### Chaotic dynamical systems

As a more complex case we investigated two Lorenz systems with non-linear (quadratic) coupling and potential self-feedback according to:
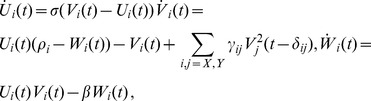
(32)with 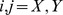 (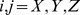 for teat case IX); parameters as indicated in table 2 for test cases IV-IX; 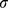, 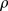 and 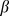, are the *Prandtl number*, the *Rayleigh number*, and a geometrical scale; 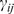 represent the coupling strengths from system 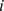 to 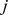, with 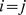 indicating (delayed) self-feedback. Note that always 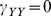 (no self-feedback 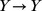). The 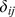 are the respective delays of the coupling or of the self-feedback. Numerical solutions to these differential equations were computed using the 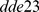 solver in MATLAB and results were resampled such that the delays amounted to the values given in table 2. For analysis purposes we analyzed the 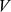-coordiantes of the systems.

#### Noisy Lorenz systems

While the proof for our approach holds strictly only for noise-free systems, in practice the proposed procedure works well for the noise profile encountered in many technical or life-sciences applications. To demonstrate this, we simulated coupled Lorenz systems as in equation 32 and afterwards added independent,Gaussian, white observation noise of varying amplitude according to:
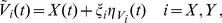
(33)where 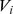 was simulated as above, 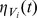 was unit variance Gaussian white noise and 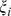 chosen such that 1%, 2% and 9% of the final signal variance were contributed by noise.

#### Ring of Lorenz systems

We also coupled three Lorenz systems into a uni-directional ring using equations 32 above, however, this time setting 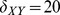, 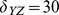, 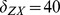, and 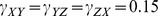.

#### Electrophysiological data

In the last test case we used data which were recorded from the turtle (Pseudemys scripta elegans) to determine interaction delays between brain areas. This experiment was described previously in [42].

#### Preparation

Experiments were approved by the German local authorities (Regierungspraesidium, Hessen, Darmstadt). One turtle (Pseudemys scripta elegans) was anesthetized with 15 mg Ketamine, and 2 mg Medetomidinhydrochloride and decapitated. The entire brain with the eyes attached was removed as described in [63]. The brain was placed in a petri dish and superfused with oxygenated ringer. The ringer consisted of (in mM) 96.5 NaCl, 2.6 KCl, 2.0 MgCl2, 31.5 NaHCO3, 20 D-glucose, 4 CaCl2 at pH 7.4 and was administered at room temperature (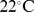).

#### Electrophysiological recordings

The electroretinogram was recorded with a chlorided silver wire in a Vaseline well that was built around the right eye. The contralateral tectal signal was recorded in a superficial layer at the center of the left tectum with a quartz/platinum-tungsten electrode (Thomas Recordings, Giessen, Germany) with impedance 1 M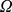 at 1 kHz. Data were amplified and filtered (1 Hz to 6 kHz) before being digitized at 32 kHz. For the analysis, the continuous data were low-pass filtered with 240 Hz, down-sampled to 500 Hz and cut into 60 trials with 50 s each.

#### Visual stimulation

A sequence of red LED light pulses with random duration (uniform distribution between 1 ms and 2 s) and random inter pulse interval (uniform distribution between 1 ms and 5 s) was triggered via the parallel port using MATLAB and the Psychophysics Toolbox extension [64], [65]. A light guide projected the full field flashes onto the retina.
